# The use of Vocalizations of the Sambirano Mouse Lemur (*Microcebus sambiranensis*) in an Acoustic Survey of Habitat Preference

**DOI:** 10.1007/s10764-017-9977-6

**Published:** 2017-07-19

**Authors:** Dan Hending, Marc Holderied, Grainne McCabe

**Affiliations:** 1 School of Biological Sciences, The University of Bristol, Bristol, BS8 1TH UK; 2 Bristol Zoological Society, Clifton, Bristol, BS8 3HA UK

**Keywords:** Acoustic survey, Habitat, *Microcebus Sambiranensis*, Mouse lemurs, Vocalization, Vocal repertoire

## Abstract

Primate vocalizations convey a variety of information to conspecifics. The acoustic traits of these vocalizations are an effective vocal fingerprint to discriminate between sibling species for taxonomic diagnosis. However, the vocal behavior of nocturnal primates has been poorly studied and there are few studies of their vocal repertoires. We compiled a vocal repertoire for the Endangered Sambirano mouse lemur, *Microcebus sambiranensis*, an unstudied nocturnal primate of northwestern Madagascar, and compared the acoustic properties of one of their call types to those of *M. murinus* and *M. rufus*. We recorded vocalizations from radio-collared individuals using handheld recorders over 3 months. We also conducted an acoustic survey to measure the vocal activity of *M. sambiranensis* in four forest habitat types at the study site. We identified and classified five vocalization types in *M. sambiranensis*. The vocal repertoires of the three *Microcebus* species contain very similar call types but have different acoustic properties, with one loud call type, the whistle, having significantly different acoustic properties between species. Our acoustic survey detected more calls of *M. sambiranensis* in secondary forest, riparian forest, and forest edge habitats, suggesting that individuals may prefer these habitat types over primary forest. Our results suggest interspecific differences in the vocal repertoire of mouse lemurs, and that these differences can be used to investigate habitat preference via acoustic surveys.

## Introduction

Acoustic communication in animals carries a variety of information to conspecifics regarding predators, mating opportunities, and presence of food (Obrist 1995). Primate vocal communication is of particular interest because primates exhibit a variety of social systems and life histories that may influence vocal repertoire, and they communicate over both short and long distances (Marler and Mitani 1988). The comparative analysis of acoustic features of primate vocalizations can reveal phylogenetic relationships and is useful in the study of vocal evolution (Gamba *et al.* 2012). Examining the bioacoustic structure of primate communications can be a useful, inexpensive, and noninvasive method to diagnose sibling species (Braune *et al.* 2008; Zimmermann *et al.* 2000), particularly for cryptic species. Cryptic primates are often solitary so they must vocalize to find potential mates, and be able to discriminate between conspecifics, as well as between biologically and vocally similar members of sympatric cryptic species (Braune *et al.* 2008). Species-specific acoustic communication can help animals to communicate successfully with conspecifics and to avoid the risk of breeding with other species.

An understanding of species-specific vocal repertoires can also be used to develop remote population monitoring tools for species-specific conservation (Gamba and Giacoma 2007). If a species can be identified through its vocal acoustic properties, bioacoustic surveys can be used to investigate species-specific vocal activity (Terry *et al.* 2005). Bioacoustic surveys are a noninvasive method of obtaining vocalization activity data and have been used successfully in studies of birds (Baldo and Mennill 2011; Dawson and Efford 2009), bats (Hackett *et al.* 2011; Obrist 1995), frogs (Love and Bee 2010; Penny *et al.* 2014), and marine habitats (Jolly and Hampton 2011). Such surveys are ideal for investigating animal habitat preference, population distributions within tropical forests, and the influence of habitat on animal vocal repertoires (Drew 2014). Different habitats have different sound transmission properties owing to their varying vegetation structures and microclimates (Wiley and Richards 1982). Changes in habitat structure alter sound transmission properties, which can lead to changes in the acoustic properties of vocalizations over very short or evolutionary periods of time (Slabbekoorn and Smith 2002). Acoustic surveys of primate calls have been used in studies of galagos (Masters 1991), tarsiers (Nietsch and Kopp 1998), and diurnal South American primates (Whitehead 1995). Although they have potential to analyze primate habitat preference, they have not yet been used to do so*.* Acoustic surveys have the potential to aid in the conservation of primate habitats, all of which are severely threatened by deforestation and habitat degradation (Ganzhorn 1987; Jolly and Sussman 2006; Schwitzer *et al.* 2011).

Many new species of cryptic, nocturnal primates, including lemurs (Mittermeier *et al.* 2010), lorises (Munds *et al.* 2013), and galagos (Masters and Couette 2015), have been described in the past 10 years and their sociobiology is poorly studied (Rode-Margono *et al.* 2016). Several of these species are almost morphologically identical, posing a problem for species-specific studies and conservation management (Henry 1985). For example, the mouse lemur genus, *Microcebus*, contains 24 species of nocturnal cryptic primates that are widely distributed throughout the forest habitats of Madagascar, often with high population densities (Kappeler and Rasoloarison 2003; Radespiel 2006). All 24 species diverged from one another recently as a result of genetic divergence in isolated subpopulations (Hotaling *et al.* 2016; Mittermeier *et al.* 2008). This accelerated speciation was caused by habitat fragmentation and natural barriers such as river catchments and extreme topography that the small-bodied lemurs are unable to cross (Wilme *et al.* 2006). Vocal fingerprinting is an effective means to discriminate between mouse lemurs (Zimmermann *et al.* 2000). For example, the acoustic properties of calls of *M. murinus* and *M. rufus* differ significantly, and this difference may be influenced by habitat (Zimmermann *et al.* 2000). No acoustic surveys have been conducted to investigate the specific habitat preference or population distribution of mouse lemurs.

We compiled a vocal repertoire for the Endangered *Microcebus sambiranensis*, or Sambirano mouse lemur, one of the smallest *Microcebus* species, which inhabits two isolated forests in northwestern Madagascar (Randriatahina *et al.* 2014; Rasoloarison *et al.* 2000). We compared this species’ vocal acoustic properties to the calls of two closely related species: *M. murinus* and *M. rufus*. We hypothesized that vocalizations of the three species will differ in their acoustic properties, owing to their different habitats. In parallel, we used an acoustic survey of *M. sambiranensis* vocal activity to investigate the species’ use and preference of different forest habitats at the study site. We hypothesized that the frequency of acoustic call detection would vary between the selected habitat types. We predicted that we would detect a higher frequency of acoustic calls in habitats composed of predominantly secondary growth with denser vegetation because these habitat types would provide the vulnerable, small-bodied mouse lemurs with better protection and concealment from predators such as owls and snakes (Perret 1998), and are therefore likely to have a higher population density of *M. sambiranensis*.

## Methods

### Study Site

**Fig. 1:**
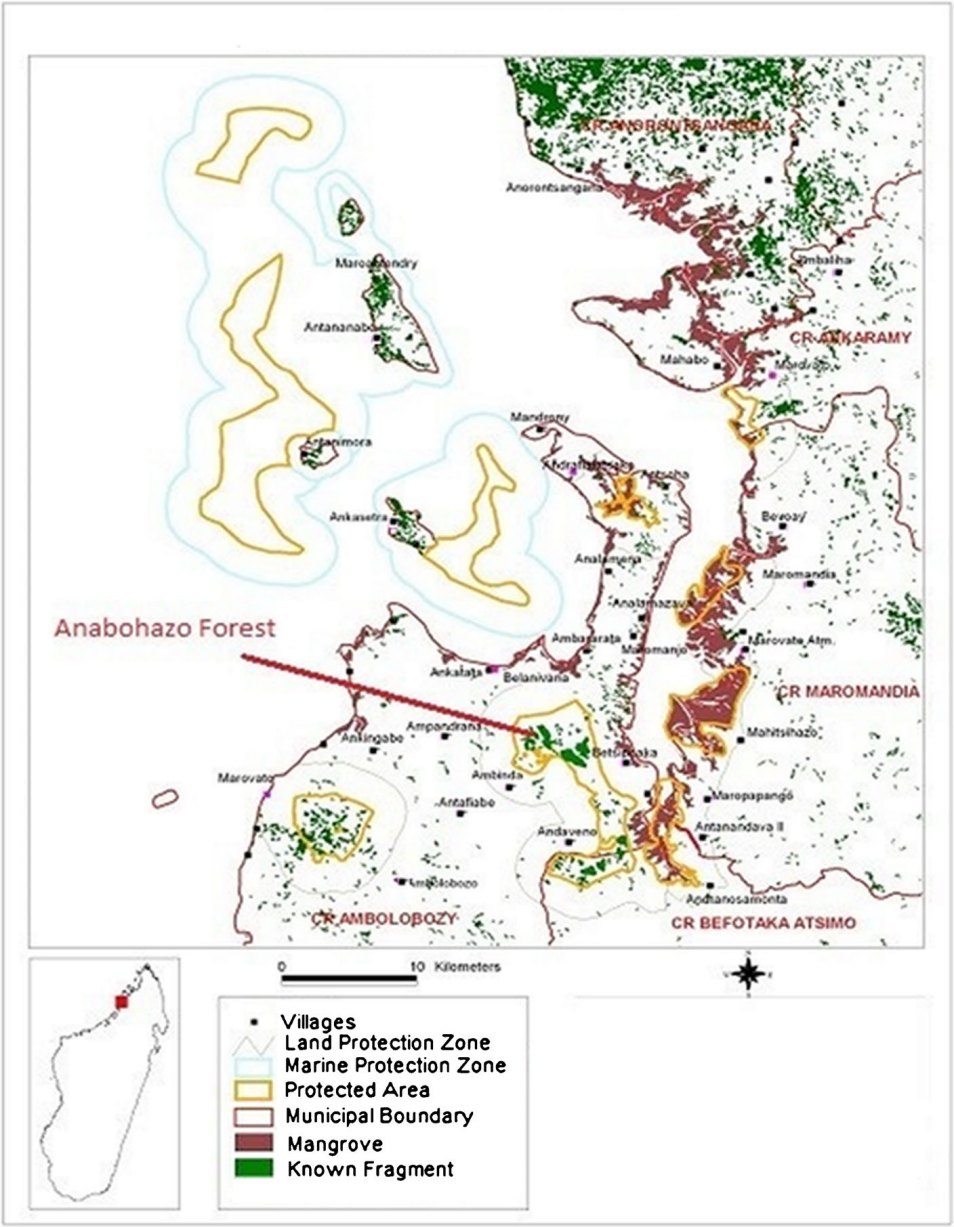
Location of Anabohazo in Sahamalaza-Iles Radama National Park, northwestern Madagascar. (Adapted from Wildlife Conservation Society Madagascar 2006).

The Anabohazo Forest is in the northeast sector (S14°19′, E47°54′) of Sahamalaza-Iles Radama National Park (Fig. 1) in the Sofia region of northwestern Madagascar (Seiler *et al.* 2013). Sahamalaza has been a UNESCO Biosphere Reserve since 2001 and was given National Park status in 2007 (Volampeno *et al.* 2015). Managed by Madagascar National Parks (MNP), the protected area of the Sahamalaza-Iles Radama National Park extends between S13°52′ and S14°27′ and E45°38′ and E47°46′ (WCS/DEC 2002). Sahamalaza-Iles Radama National Park lies within the Sambirano domain of Madagascar, a transitional area between the rainforests of the north and the dry, deciduous forests of the west (Project ZICOMA 1999), where the climate is hot and subhumid (Andreone *et al.* 2001). There is a hotter, wet season from November until April with a mean temperature of 32.0 °C followed by a cooler, dry season from May until October with a mean temperature of 20.6 °C (Schwitzer *et al.* 2007a, b). The mean precipitation for the area is 1750 mm of rainfall (Project ZICOMA 1999), most of which falls in the wet season. Anabohazo is a semihumid forest, characterized by rolling hills of ca. 300–350 m a.s.l. that are intersected by small, seasonal streams (Andreone *et al.* 2001). The total area of the Anabohazo forest is 5275 ha, the largest of the three forest blocks remaining on the peninsula, the others being Ankarafa and Analavory (Randriatahina *et al.* 2014). The vegetation is characteristic of the western dry forests of Madagascar but there are many tree species here that are unique to the Sambirano domain (Birkinshaw 2004).

Sahamalaza-Iles Radama National Park harbors eight different lemur species, including the last remaining populations of the Critically Endangered blue-eyed black lemur, *Eulemur flavifrons* and the Sahamalaza sportive lemur, *Lepilemur sahamalazensis* (Schwitzer *et al.* 2013). Other lemur species found here are the Endangered northern giant mouse lemur, *Mirza zaza*, and aye-aye, *Daubentonia madagascariensis*; the Vulnerable black lemur, *Eulemur macaco* and western bamboo lemur, *Hapalemur occidentalis*; and the fat-tailed dwarf lemur, *Cheirogaleus medius.* The presence of *Microcebus sambiranensis* was first confirmed in the National Park in 2014 (Randriatahina *et al.* 2014). Lemur populations here are threatened by habitat destruction due to slash-and-burn agriculture, in addition to selective logging and mining (Seiler *et al.* 2010).

### Reference Vocal Collection

We captured five male and three female *Microcebus sambiranensis* individuals by hand and using 10 live-traps (LFG Folding Trap, Sherman, Tallahassee, FL, USA) over 4 days at the start of the study, March 23, 2015– March 26, 2015. We set the traps nightly at 18:00 h and checked them every hour until 02:00 h. We logged GPS waypoints of the capture site trees. We anesthetized captured individuals with 10 mg/kg of Telazol® anesthetic; identified their sex; and recorded their head length, body length, tail length, and total body mass using Vernier callipers and spring scales. We collared the captured individuals with TW4 PiP light-weight collars (BioTrack, Wareham, UK; frequency range 150.061–150.508). A trained, experienced veterinarian secured collars around the subject’s neck using a zip tie and tightened it so that accidental asphyxiation did not occur. We cut off any remaining exposed length of cable tie using scissors. Once the collared *M. sambiranensis* individuals had fully recovered from the effects of the anesthetic, a recovery time of ca. 8 h, we released them at the tree in which they were captured.

We tracked radio-collared individuals using a portable radio receiver (Regal 2000, Titley Scientific, Columbia, MO, USA) with a two-element antenna (Titley Scientific, Columbia, MO, USA) during night observations (18:00–04:00 h) from March 27, 2015 to May 31, 2015. We collected 350 h of night observations, tracking one lemur per night. We recorded any vocal activity of the collared lemurs using a portable handheld recorder (R-05, Roland, Hamamatsu, Japan) equipped with an internal stereo microphone set to a sampling rate of 96 kHz, and saved recordings as 320 kbps WAV audio files. We set the sampling rate to 96 kHz because some known vocalizations of mouse lemurs go into the ultrasound frequency range (Braune *et al.* 2005). The handheld recorder had a 128 × 64 dot graphic display screen that indicated any nearby sound up to 48 kHz detected by the internal microphone, even if the microphone was not recording. This allowed us to identify when our collared individuals were vocalizing in the ultrasound range, enabling us to record the higher frequency vocalizations although we could not hear them ourselves. We used focal observations of the vocalizing individuals to determine the context in which the vocalization was being used. We noted any interactions with other mouse lemurs, responses to stimuli such as predator presence and specific behaviors if they corresponded to or triggered a vocalization, a method previously used to determine call context in other mouse lemurs (Braune *et al.* 2005; Scheumann *et al.* 2007).

### Reference Vocal Extraction

To compile the vocal repertoire, we selected only the highest quality vocalizations with a low level of background recording noise from the 96-kHz sampling rate handheld recorder data. We used SASLab Pro 5.2.07 (Avisoft Bioacoustics, Berlin, Germany) to filter out frequencies of background noise; weather disturbance such as wind and rain; and the loud calls of other animals such as bats, frogs, insects, and zebu cattle. We used a finite impulse response (FIR) filter, a method successfully used by Love and Bee (2010). We used a high-pass FIR filter for vocalizations at a frequency of 15 kHz or more, with a cutoff of 14 kHz. For lower frequency vocalizations, we used a low-pass FIR filter with a cutoff frequency of 15 kHz. For any vocalizations with a fundamental frequency of around 14–15 kHz, we used a low-pass FIR filter with a cutoff frequency of 20 kHz. We also analyzed all recorded vocalizations by ear at a lower playback frequency, 22.1 kHz, to bring them fully into the human auditory frequency range. We normalized the vocalization amplitude to 90% of the recording range, and deleted any remaining unwanted background noise manually using the spectrogram eraser function. We then analyzed the acoustic properties of individual vocalization units to preclassify our recorded vocal types. We preclassified vocalization types according to a pilot acoustic study of gray mouse lemurs, *Microcebus murinus*, at Bristol Zoo Gardens (D. Hending, *unpubl. Data*) and to similar vocalizations published for other mouse lemur species (Braune *et al.* 2005; Cherry *et al.* 1987; Glatston 1979; Leliveld *et al.* 2011; Zimmermann *et al.* 2000). We defined vocalizations as a single unit if call units were separated from each other by a silence more than double the call unit’s duration, and multiunit if the call interval was less than double the unit’s duration (Rasoloharijaona *et al.* 2006). We used the automatic parameters measurement function in SASLab Pro to create a table of temporal and frequency parameters from a DDE parameter file for frequently recorded vocalizations (Table I).

**Table I:** Descriptions of acoustic parameters measured from spectrograms of *Microcebus sambiranensis* vocalizations recorded in Anabohazo forest, northwest Madagascar (March 27, 2015–May 31, 2015), using the automatic parameters measurement function in SASLab Pro

Acoustic Parameter	Description
Duration (s)	Time between the onset and offset of a call unit
Interval (s)	Time between the offset of one unit and the onset of the next unit
Disttomax (s)	Time between the onset of the unit and the unit’s point of maximum frequency
Mean peak frequency (Hz)	Mean frequency at maximum amplitude during the unit’s total duration
Mean minimum frequency (Hz)	Mean minimum frequency during the unit’s total duration
Mean maximum frequency (Hz)	Mean maximum frequency during the unit’s total duration
Mean bandwidth (Hz)	Range between the mean minimum and mean maximum frequencies for the total unit duration
Start peak frequency (Hz)	Frequency at maximum amplitude of the unit start
Start minimum frequency (Hz)	Minimum frequency at the onset of the unit
Start maximum frequency (Hz)	Maximum frequency at the onset of the unit
Start bandwidth (Hz)	Range between maximum and minimum start frequency
End peak frequency (Hz)	Frequency at maximum amplitude of the unit end
End minimum frequency (Hz)	Minimum frequency at the offset of the unit
End maximum frequency (Hz)	Maximum frequency at the offset of the unit
End bandwidth (Hz)	Range between maximum and minimum end frequency

### Species Comparison

We compared six acoustic parameters of one call type, the whistle, between three mouse lemur species using the acoustics parameters of Whistle Type 1 for *Microcebus sambiranensis* from this study and data harvested from the published results of Zimmermann *et al.* (2000) for *M. murinus* and *M. rufus*. We used the means, standard deviations, and sample sizes for each variable as we had no raw data for *M. murinus* and *M. rufus.*

### Selection of Acoustic Survey Habitats

**Fig. 2:**
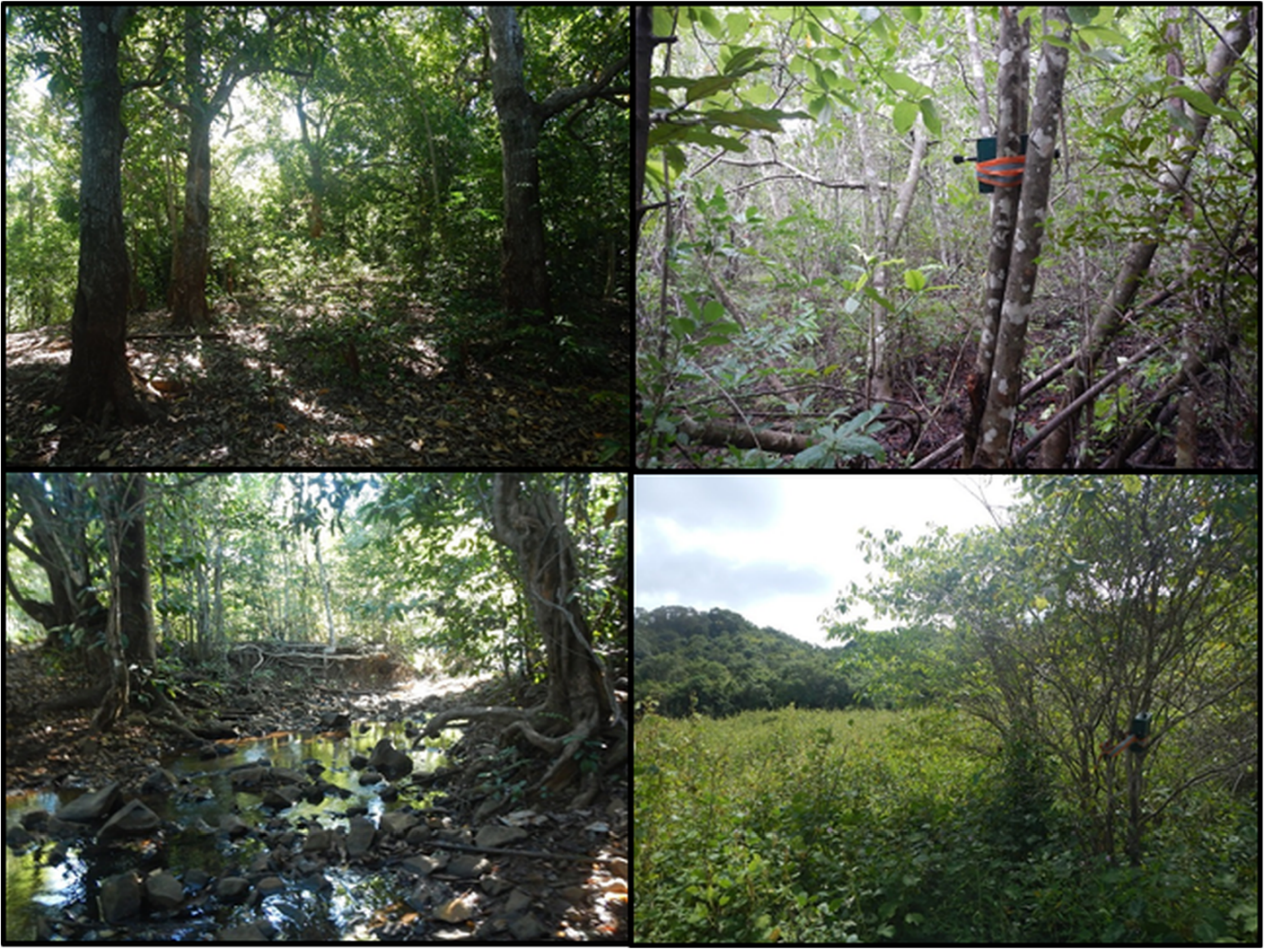
Habitat types selected for acoustic surveying of *Microcebus sambiranensis*, March 1, 2015–May 31, 2015 in Anabohazo forest, northwest Madagascar. Clockwise from top left: Primary forest, secondary forest, forest edge, and riparian forest. The acoustic recorder can be seen in secondary forest and forest edge.

We selected four habitat types for acoustic surveys: (1) primary forest, (2) secondary forest, (3) riparian forest, and (4) forest edge, including the boundary of large vegetation clearings (Fig. 2). We characterized each habitat using preliminary vegetation plot data (F. Ramiadantsihoarana *unpubl. Data*). We characterized primary forest as areas of undisturbed forest dominated by sparsely distributed, old-growth trees with a mean diameter at breast height (DBH) of ≥200 mm; secondary forest as areas of densely vegetated juvenile or subadult trees with a mean DBH of <200 mm; riparian forest as an area of forest within and not more than 10 m away from a flowing water source or dry stream bed; and forest edge/boundary as an area of forest within and not more than 10 m away from a large open area.

We then selected five different sites for each habitat type, resulting in a total of 20 sites for acoustic recording. We selected the order of acoustic monitoring at each site at random and positioned the recording equipment ≥50 m away from human-made paths used by local villagers to access neighboring villages. This was to minimize human-produced noise recorded and human interference. The 20 chosen sites were spread through 80 ha of the study site: 1.5% of the total area of Anabohazo forest, and over an altitude of 245–359 m.

### Acoustic Habitat Surveys

We conducted acoustic surveys from March 1, 2015 until May 31, 2015, the transitional phase between the wet and dry season, using an automated terrestrial wildlife acoustic recorder (Song Meter SM2+, Wildlife Acoustics, Maynard, MA, USA) equipped with two weather-proof microphones (SMX-II, Wildlife Acoustics, Maynard, MA, USA). We attached the acoustic recorder to a tree at the recording site at a height of 1.0–1.5 m above the ground so that the chance of device tampering by rats and other small ground-dwelling animals was minimal, an approach used by Hackett *et al.* (2011). The acoustic recorder produced stereo files at a 48-kHz sampling rate, 16-bit resolution, resulting in 320 kbps WAV audio files, an approach that has worked successfully in other acoustic surveys of terrestrial organisms (Baldo and Mennill 2011). We chose a 48-kHz sampling rate because we expected it to be sufficiently high to record the fundamental harmonic of all *M. sambiranensis* vocalizations in the vicinity of our acoustic recorder. We tested and confirmed this in a pilot acoustic study of captive *Microcebus murinus* vocalizations at Bristol Zoo Gardens (D. Hending *unpubl. Data*). We recorded from 18:00 h to 06:00 h for three repeat nights at each site, a total of 60 nights worth of recording. We saved recordings as four 3-h WAV files per night.

### Acoustic Survey Vocal Extraction

We converted stereo WAV files from the acoustic survey into mono WAV files by removing the channel of the recording with the lowest recorded volume in SASLab Pro 5.2.07 (Avisoft Bioacoustics, Berlin, Germany). We split them into 10-s recordings with an accompanying spectrogram for visual analysis in MATLAB® R2014a (MathWorks, Natick, MA, USA) using the splitWAVs_batch script. In total, the 12-h recordings from the 60 nights of the acoustic survey required 263,700 ten-second audio files with spectrograms. The MATLAB® spectrograms displayed lemur vocalizations clearly and enabled us to visually check for the presence of *Microcebus sambiranensis* vocal activity. We could examine two or three spectrograms in 1 s to detect mouse lemur vocalizations. Due to our sampling rate of 48 kHz, it was possible that some of the higher frequency calls may have been only partially recorded. We only included calls that were fully, not partially, visible in our MATLAB® spectrograms for analysis of the acoustic survey results, and were therefore unable to compare the number of partially recorded calls between habitat types. Additionally, we were very conservative in our selection of calls for analysis from the acoustic survey; we only included calls with a peak amplitude at least 19 dB above the background noise level. We did not measure or analyze the acoustic properties of calls recorded in the acoustic survey and we did not include them in our vocal repertoire compilation.

### Data Analysis

We conducted statistics on recorded vocalization types in IBM SPSS 21.0 (SPSS Inc., Chicago, IL, USA). For acoustic classification of call types, we log transformed (log_e_) the highly skewed acoustic parameter data for ease of interpretation and to meet the normality assumptions of further analyses. We used principal component analysis (PCA) to condense the large set of measured variables into a smaller set of principal components. We then used stepwise discriminant function analysis (DFA) with leave-one-out cross validation of these principal components to test whether our identified, preclassified vocalization types were qualitatively classified as distinct vocal groups. We used one-way ANOVA to test for significant differences in the acoustic parameters of whistle calls between *Microcebus sambiranensis*, *M. murinus*, and *M. rufus*. For these multiple comparisons, we applied Bonferroni correction (*a* = 0.05 /*N*, where *N* is the number of comparisons). We used a univariate general linear model to suggest habitat preference by analyzing which habitat types had a significantly larger number of mouse lemur vocalizations. The dataset met the assumptions of the linear model, where the response variable was habitat type. We counted multiunit calls as one vocalization. We used an α level of 0.05 for all analyses except where we applied the Bonferroni correction.

## Ethical Note

All research complied with UK Home Office policies when working with animals and all research adhered to the legal requirements of Madagascar. Research in the Sahamalaza-Iles Radama National Park was permitted by Madagascar National Parks (Permit number 049/15/MEEF/SG/DGF/DCB.SAP/SCB). We consulted the Code of Best Practices for Field Primatology when planning all methods undertaken in this study. Capturing and collaring of the study population individuals took place under the supervision of a professional, experienced veterinarian team; details of the procedure are described in the Reference Vocal Collection section. At the end of the study, the veterinarian team recaptured all radio collared individuals and removed the radio collars using a pair of scissors. We released the individuals at the tree in which we captured them.

## Data Availability

The datasets generated and analyzed during the current study are available from the corresponding author on reasonable request.

## Results

### Vocal Repertoire and Behavioral Context

**Fig. 3:**
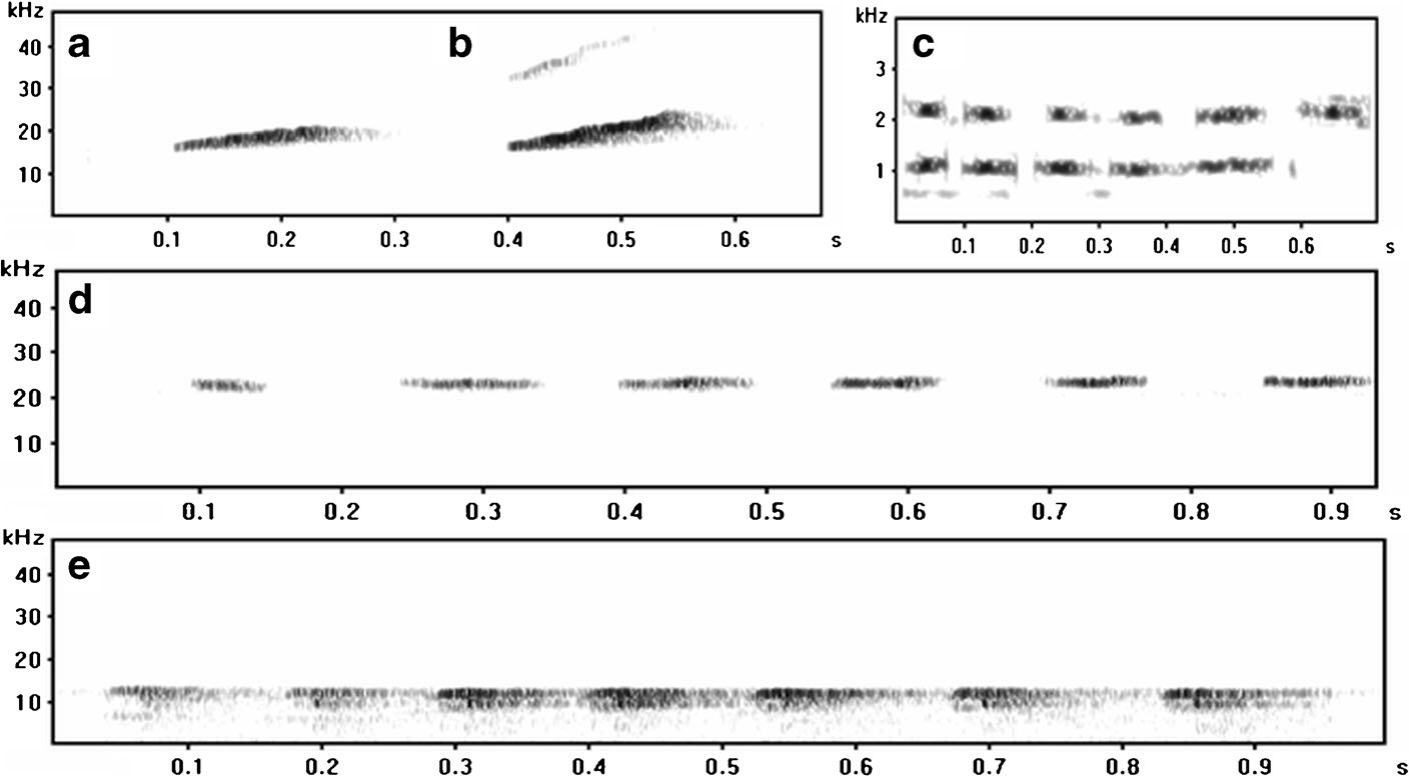
Spectrograms of the five recorded calls of *Microcebus sambiranensis* created from separate sound files, recorded in Anabohazo forest, northwestern Madagascar (March 27, 2015–May 31, 2015). **a** Whistle Type 1. **b** Whistle Type 2. **c** Purr. **d** Whistle Type 3. **e** Chitter. Generated in Avisoft SASLab Pro; FFT length: 512 points; 1024-point Hamming Window; 96 kHz sampling rate; 50% window overlap resulting in 188 Hz frequency resolution and 2.67 ms temporal resolution.

We recorded 71 mouse lemur vocalizations during 350 h of opportunistic handheld recording, a rate of 0.2 vocalizations per hour. After analyzing these recordings, we preclassified five vocal types for *Microcebus sambiranensis* based on spectral (Fig. 3) and acoustic properties (Table II)*.* Some call types were single unit and others were multiunit.

**Table II:** Acoustic parameters of five vocalization types of *Microcebus sambiranensis* recorded in Anabohazo forest, northwestern Madagascar (March 27, 2015–May 31, 2015)

Call type	Duration(s)	Interval(s)	Disttomax(s)	Mean peak frequency (Hz)	Mean minimum frequency (Hz)	Mean maximum frequency (Hz)	Mean bandwidth (Hz)	Start peak frequency (Hz)	Start minimum frequency (Hz)	Start maximum frequency (Hz)	Start bandwidth (Hz)	End peak frequency (Hz)	End minimum frequency (Hz)	End maximum frequency (Hz)	End bandwidth (Hz)
Whistle Type 1	0.162	1.436	0.060	22,100	21,000	24,000	3000	21,700	20,600	24,300	3000	23,200	21,700	27,700	6000
*N* = 20	(0.142–0.172)	(1.311–1.537)	(0.050–0.090)	(21,700–22,500)	(20,600–21,150)	(23,600–24,500)	(3000–3300)	(21,500–22,500)	(20,400–21,150)	(23,800–25,650)	(3000–4250)	(23,000–24,000)	(20,850–21,850)	(26,800–27,700)	(5200–6300)
Whistle Type 2	0.145	1.025	0.076	21,000	19,800	23,400	3000	18,900	20,800	22,650	3000	18,000	20,600	26,200	5600
*N* = 20	(0.131–0.155)	(0.916–1.119)	(0.064–0.099)	(20,900–21,800)	(18,900–20,700)	(22,725–24,300)	(3000–3300)	(18,300–19,575)	(20,200–21,300)	(22,300–22,900)	(3000–3000)	(17,200–18,300)	(20,200–21,300)	(25,725–27,000)	(5100–6000)
Whistle Type 3	0.066	0.145	0.029	22,600	22,100	23,600	1500	22,600	21,900	23,600	1600	22,800	21,500	23,600	2000
*N* = 6	(0.058–0.069)	(0.144–0.154)	(0.023–0.035)	(22,450–22,700)	(21,700–22,200)	(23,600–23,600)	(1400–1750)	(22,500–22,800)	(21,700–22,100)	(23,600–23,600)	(1450–1700)	(22,700–23,100)	(20,600–22,000)	(23,600–23,600)	(1550–2950)
Chitter	0.070	0.139	0.021	11,200	9700	13,500	3300	11,200	7500	15,000	8600	11,600	7800	15,000	7100
*N* = 13	(0.056–0.075)	(0.127–0.148)	(0.014–0.022)	(11,200–11,600)	(8800–10,100)	(13,500–13,500)	(3000–4650)	(10,650–11,200)	(5600–8000)	(14,000–15,500)	(6900–14,000)	(11,600–11,600)	(7800–8400)	(14,400–15,300)	(6700–13,800)
Purr	0.040	0.110	0.020	1560	945	2265	1295	1045	915	2295	1265	1545	900	2250	1325
*N* = 6	(0.040–0.060)	(0.110–0.120)	(0.010–0.030)	(1045–2075)	(930–960)	(2220–2303)	(1258–1333)	(1030–1083)	(900–983)	(2198–2340)	(568–1370)	(1030–2105)	(878–945)	(2220–2340)	(793–1385)

Data are medians with interquartile ranges; *N* is the number of units analyzed of each call type. Parameters measured are duration, interval between units, duration between unit onset and maximum frequency (Disttomax), mean peak frequency, mean minimum frequency, mean maximum frequency, mean bandwidth, start peak frequency, start minimum frequency, start maximum frequency, start bandwidth, end peak frequency, end minimum frequency, end maximum frequency, and end bandwidth

PCA identified four call parameters as principal components for call type discrimination, which we submitted to the DFA (Table III). DFA revealed significant differences between our five preclassified vocal types (Wilk’s λ = 0.013, *F*_56, 1592_ = 7.076, *P* < 0.001). Leave-one-out cross-validation correctly classified 96.4% of the preclassified vocalizations by call type (Table IV). All Whistle Type 1, Whistle Type 3, Chitter and Purr calls were classified correctly. Whistle Type 2 had a lower classification rate of 89.5%, with 10.5% of cases being classified as Whistle Type 1.

**Table III:** Principal component call parameters contributing to the discrimination and classification of *Microcebus sambiranensis* call types recorded in Anabohazo forest, north-western Madagascar (March 27, 2015–May 31, 2015)

Principal call components	Mean minimum frequency	Mean minimum frequency, mean maximum frequency	Mean minimum frequency; mean maximum frequency, mean bandwidth	Mean minimum frequency; mean maximum frequency; mean bandwidth, start peak frequency
Discriminant function	1	2	3	4
Variance (%)	93.0	4.9	1.7	0.4
Eigenvalue	525.420	27.829	9.703	2.245
Canonical correlation	0.999	0.983	0.952	0.832
Wilks’ λ	0.000	0.001	0.029	0.308
df	60	42	26	12
Sig.	<0.001	<0.001	<0.001	<0.001

**Table IV:** Percentages and call type numbers of call classification results from stepwise discriminant function analysis with leave-one-out cross validation analysis for calls of *Microcebus sambiranensis* recorded in Anabohazo forest, northwestern Madagascar (March 27, 2015–May 31, 2015)

Call type	Predicted call type				
	Whistle Type 1	Whistle Type 2	Whistle Type 3	Chitter	Purr
Whistle Type 1	100.0 (18)				
Whistle Type 2	10.5 (2)	89.5 (17)			
Whistle Type 3			100.0 (6)		
Chitter				100.0 (8)	
Purr					100.0 (5)

**Fig. 4:**
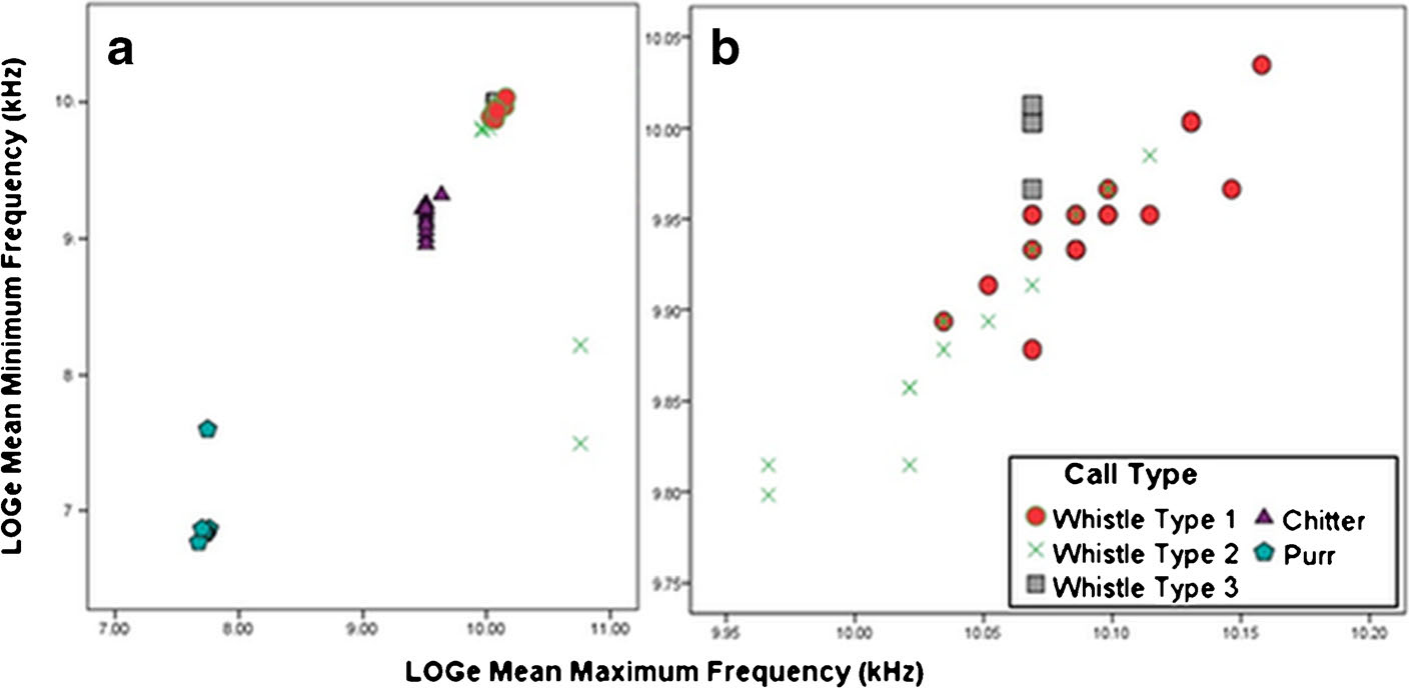
Scatterplots of the first two principal components in our DFA classification analysis for all five *Microcebus sambiranensis* call types (**a**) and a zoomed in version of the three whistle calls (**b**). All vocalizations were recorded in the Anabohazo forest, northwestern Madagascar (March 27, 2015–May 31, 2015).

Our correctly classified vocal types are distinct when plotted on a scatterplot of the first two principal components (Fig. 4a). When inspected more closely, the three whistle calls spread out slightly despite their similar acoustic properties and spectrographic appearances (Fig. 4b).

We observed Whistle Type 1 the most frequently of the five vocalizations. It is a tonal, single-unit loud call where the frequency rises at a shallow gradient throughout the duration of the call. The call is a single piercing unit and is always used in a repeated sequence. The frequency of the call increases by 2.7 kHz between the onset and offset (Table II). We always observed the call being emitted by a lone adult attempting to communicate with other members of the population, with no obvious threat, over a long distance.

We observed Whistle Type 2 on three occasions, where it was repeated for at least half an hour. Like Whistle Type 1, it is a tonal, piercing single unit loud call with a rising frequency throughout the call unit. Figure 3b shows that this call is very similar to Whistle Type 1 (Fig. 3a) but has a harmonic overtone at a frequency of 30–44 kHz and a steeper rise in frequency throughout the call unit. Its acoustic properties are similar to those of Whistle Type 1 (Table II). However, we observed this call being used by adults only when a potential threat was nearby such as a snake in the caller’s tree or an owl in the immediate vicinity. Therefore, it may function as an alarm call or a distress signal. We recorded all Whistle Type 2 calls during the final 3 weeks of the field season; the collared individuals were well habituated by this time and it is unlikely that any distress call was due to the presence of a researcher.

The Purr is a soft multiunit call with a frequency of around 1 kHz. It has a second harmonic overtone that is double the frequency (Table II, Fig. 3c). We observed it on one occasion between two adults with overlapping home ranges during allo-grooming. It is similar spectrographically to the *Microcebus murinus* Purr detailed in Glatston (1979).

We heard Whistle Type 3 once, from a lone adult. Unlike the other two whistle type calls described here it is multiunit. The Chitter call is also a multiunit, tonal call that was used between two members of the population over a short distance.

The mean acoustic parameters of Whistle Type 1 were all significantly different between *M. sambiranensis*, *M. murinus*, and *M. rufus* (Table V). When Bonferroni correction was applied to our ANOVA analysis (*a* = 0.05/6; *a* = 0.003), the results for all six comparisons were still statistically significant (*P* < 0.001).

**Table V:** Comparison of the acoustic parameters of Whistle Calls in three mouse lemur species

Whistle calls							
Species	*M. sambiranensis*		*M. murinus*		*M. rufus*		ANOVA
	Mean	SD	Mean	SD	Mean	SD	
Mean start frequency (kHz)	20.7	± 0.60	14.3	± 1.60	18.3	± 3.40	df = 2, *F* = 54.544, *P* < 0.001
Mean end frequency (kHz)	23.4	± 1.00	14.3	± 2.00	17.4	± 3.90	df = 2, *F* = 72.401, *P* < 0.001
Mean minimum frequency (kHz)	20.7	± 0.75	13.8	± 1.70	17.2	± 3.80	df = 2, *F* = 48.042, *P* < 0.001
Mean maximum frequency (kHz)	27.1	± 1.24	15.6	± 1.50	18.7	± 3.40	df = 2, *F* = 158.328, *P* < 0.001
Mean duration (s)	0.158	± 0.02	0.128	± 0.05	0.084	± 0.06	df = 2, *F* = 14.382, *P* < 0.001
Interval (s)	1.433	± 0.13	0.223	± 0.07	0.127	± 0.06	df = 2, *F* = 1646.568, *P* < 0.001

Vocalizations of *Microcebus sambiranensis* were recorded in the Anabohzo forest, northwestern Madagascar (March 27, 2015–May 31, 2015). Data for *Microcebus murinus* and *M. rufus* are from Zimmermann *et al.* (2000), where all vocalizations were recorded at the Institute of Zoology, University of Veterinary Medicine Hanover (1990–1998)

### Acoustic Survey of Habitat Preference

We recorded 27 vocalization instances for *Microcebus sambiranensis* during 720 h of acoustic survey recording, a rate of 0.05 successful recordings per survey hour. There was a significant difference in mouse lemur call presence between habitat types (general linear model: *F*_3,14_ = 16.000, *P* < 0.001). We recorded significantly more mouse lemur calls in secondary forest (9, *F*_1,3_ = 5.559, *P* = 0.033), riparian forest (6, *F*_1,3_ = 6.000, *P* = 0.028), and forest edge (12, *F*_1,3_ = 56.000, *P* < 0.001) compared to primary forest (0).

## Discussion

We identified and correctly classified five distinct vocal types for *Microcebus sambiranensis* that were similar in acoustic structure and function to calls of other mouse lemur species. We named these calls according to the corresponding call types in the vocal repertoires of the closely related *M. murinus* and *M. rufus* (Glatston 1979; Zimmermann *et al.* 2000). Although two of the vocal types, Whistle Type 1 and Whistle Type 2, were clearly related and possessed similar properties, we found a significant statistical difference in their acoustic structure, so we propose them as separate call types. We found species differences in the spectrograms of the call types. For example, the Purr call of *M. sambiranensis* is at a lower frequency than that of the *M. murinus* Purr call (Glatston 1979) and both the Whistle Type 1 and Whistle Type 2 calls of *M. sambiranensis* are at higher frequencies than whistle calls of *M. murinus*. There are also similarities in the whistle calls of different species. *M. sambiranensis* and *M. murinus* whistle calls have rising frequencies throughout the call (Glatston 1979; Zimmermann *et al.* 2000). The Whistle Type 3 call of *M. sambiranensis* is similar to some of the short whistle calls described for *M. murinus* and *M. rufus* by Zimmermann *et al.* (2000), as it is multiunit with interunit intervals that are less than double the duration of the call’s units. Additionally, the Chitter call of *M. sambiranensis* is very similar in spectrographic appearance to the Chitter call described for *M. murinus* (Glatston 1979).

We found significant differences among the three species in all the acoustic parameters we tested for whistle calls. Our spectrogram analysis of the other call types described here for *Microcebus sambiranensis* also differ from spectrograms of the same call types in *M. murinus* and *M. rufus* (Glatston 1979; Zimmermann *et al.* 2000). These results support our hypothesis that the vocal repertoires of the three mouse lemur species have distinctive acoustic parameters. We did not perform statistical comparisons of the acoustic properties of the other call types described here for *M. sambiranensis* and the same calls in *M. murinus* and *M. rufus* because our sample sizes of high-quality recordings were too small for Whistle Type 3 (*N* = 6) and the Purr (*N* = 6). Also, there are no quantitative acoustic data on the Chitter call of other mouse lemur species, so statistical comparisons between the three mouse lemur taxa for this call type was not possible.

The findings of our acoustic survey suggest that *M. sambiranensis* prefers secondary growth habitats with younger trees, although we cannot rule out habitat differences in detection. We recorded no mouse lemur vocal activity in the primary forest sites. *M. sambiranensis* may avoid primary forest because the thick branches of old growth trees expose them to attack from nocturnal predators such as owls and snakes (Perret 1998). Secondary forest has denser growth that offers better protection for the small-bodied mouse lemurs. The canopy in the primary forest was well in excess of 20 m and vocalizations of mouse lemurs that may have been within the primary forest canopy would not have carried far enough to have been picked up by our acoustic recorder. Thus *M. sambiranensis* may use primary forest but we were unable to confirm this with our acoustic survey. Also, we did not visually observe any *M. sambiranensis* in primary forest habitat.

Our recording rates of 0.05 recordings per hour suggest that acoustic surveys are not an efficient way to collect large quantities of mouse lemur calls for vocal repertoire compilation or to investigate vocal activity patterns. Radio-tracking and recording calls of collared mouse lemurs produced better results with more than double the vocal recordings in half the recording time. The acoustic survey recording rate was likely affected by the position of the recording equipment. For example, if a mouse lemur vocalized from high in a canopy it is likely that the acoustic recorder would not have recorded the call as it would have been too far away. Additionally, the calls of mouse lemurs are comparatively low amplitude compared to the calls of other forest animals and the acoustic recorder would have to be in close proximity of the individual emitting the call. These factors should be taken into account in future acoustic surveys of cryptic primate vocal activity.

Further research, with some methodological adjustments, is needed to expand our knowledge of this species’ vocal repertoire. Our equipment in this study limited our sampling rate to a maximum of 96 kHz, meaning that we lost some of the upper harmonics in our recordings. In future studies, we will use equipment capable of a sampling rate double the highest target frequency for mouse lemurs, i.e., a sampling rate of 200 kHz (Braune *et al.* 2005), which will ensure the upper harmonics are not cut from our recordings.

There have been many studies of the vocalizations of *Microcebus murinus* both in situ and ex situ. The vocal repertoire of *M. murinus* contains more call types than those described here for *M. sambiranensis* (Glatston 1979), suggesting that the repertoire presented here is not comprehensive. Data collection for this study took place outside the suspected mating season of this species (September to December), the period leading up to and including the first part of the wet season (Radespiel 2006); thus, it is likely that calls used in mating and associated social contexts, such as the Trill (Leliveld *et al.* 2011) are missing from the repertoire we describe. We also observed very few social and agonistic interactions, so we probably missed associated vocalizations, such as the Grunt and Tsak calls observed *in M. murinus* (Leliveld *et al.* 2011). Other call types described for other *Microcebus* species are infant specific or used between a mother and her offspring, such as the Infant Distress Call, Elimination Call, and the Whitter (Glatston 1979). We did not record these classes of calls as we did not observe any infants. Finally, the *M. murinus* Disturbance Call has been observed only in captivity and we found nothing similar in this study (Glatston 1979).

The understanding of species-specific acoustic communication is important for the field identification of cryptic, nocturnal primate species and has valuable implications for conservation (Seiler *et al.* 2015). The European Association for the Study and Conservation of Lemurs (AEECL) and Mikajy Natiora, two nongovernmental organizations active in Madagascar that endeavour to protect several Endangered lemur species, use the scientific understanding of species habitat use to target specific forest habitat types with their long-term reforestation program. This study will be used to inform these reforestation efforts to restore degraded habitats that are important for the survival of *Microcebus sambiranensis*. The information provided in this study will also help these nongovernmental organizations to select locations for long-term monitoring of the Sambirano mouse lemur. Additionally, they can use loud call acoustic surveys, such as the one used in this study, to estimate population densities and develop low-cost, long-term remote acoustic sampling tools. These will act as remote acoustic sampling tools to assess the population distribution of the species, as there are currently no conservation methods in place for this Endangered species (Andriaholinirina *et al.* 2014).

Most *Microcebus* species are classified as either Endangered or Critically Endangered and most are unstudied (Andriaholinirina *et al.* 2014). To further the scientific understanding of mouse lemur acoustic communication, we need to compile vocal repertoires of unstudied *Microcebus* species to explore the phylogenetic and ecological factors that shape them. For example, we can ask whether the vocal repertoires of more closely related species are more similar in their acoustic properties than more genetically distinct species, and to what extent environmental factors influence mouse lemur vocal repertoires. These factors are particularly important to consider because vocal behavior can reveal the genetic fitness and adaptability of a species (Terry *et al.* 2005).

## Conclusion

We described five distinct call types for *Microcebus sambiranensis* relating to communication over short and long distances and distress. The acoustic parameters of one call type, Whistle Type 1, differed significantly from those described for other mouse lemur species. An acoustic survey revealed that *M. sambiranensis* may prefer secondary growth habitats with denser vegetation over old-growth primary forest. Despite the acoustic survey’s success in suggesting the habitat preference of *M. sambiranensis*, it was not an efficient method to record large quantities of mouse lemur calls for vocal repertoire compilation, although it is a useful, noninvasive method to explore the distribution and habitat preferences of a mouse lemur species in a study site. The acoustic traits of the *Microcebus* genus remain poorly understood, despite their potential to reveal the genetic fitness and environmental adaptability of a species. Knowledge of vocal communication can reveal ecological and social traits of species that are important for the development of conservation methods. The *Microcebus* genus is threatened with extinction. Compiling vocal inventories for more species would allow us to further unravel the question of how phylogeny and environmental influences shape their methods of acoustic communication.
